# Adaptation and Validation of the Perceived Control in Unemployment Scale

**DOI:** 10.3389/fpsyg.2019.00383

**Published:** 2019-02-28

**Authors:** Claude Houssemand, Raymond Meyers, Anne Pignault

**Affiliations:** ^1^ECCS, Institute for LifeLong Learning and Guidance, University of Luxembourg, Esch-sur-Alzette, Luxembourg; ^2^Psychology & Neuroscience Laboratory, University of Lorraine, Nancy, France

**Keywords:** unemployment, locus of control, perceived control, job search, scale

## Abstract

Perceived control plays an important role in the understanding of people's experiences with unemployment and reemployment. Yet, no scale has been designed specifically to measure people's perceived control in an unemployment situation. In the current study, using two independent samples with 1,009 and 831 unemployed people in France and Luxembourg, respectively, we created and tested a three-dimensional Perceived Control in Unemployment Scale that was based on Levenson's (1973, 1981) theory. An exploratory factor analysis (Study 1) and a confirmatory factor analysis (Study 2) showed that the data were consistent with the theoretically postulated three-factor model. In addition, we established convergent and discriminant validity with several adaptive and non-adaptive dimensions in two independent samples of 141 unemployed people and 384 recently unemployed people in Luxembourg (Studies 3 and 4, respectively). Perceived control did not change over a period of 6 months of unemployment, yet the three types of perceived control measured at the beginning of unemployment predicted employment status 6 months later. Unemployed people with perceptions of internal control or control from powerful others found jobs more quickly, whereas the perception that chance was the controlling factor predicted longer unemployment.

In recent decades, a large body of research in psychology has focused on the concept of Locus of Control (LOC). Generally considered a personality trait that determines the extent to which people believe that the rewards they receive in life can be controlled by their own personal actions or are external to themselves (Rotter, 1966; Lefcourt, 1982), LOC can be defined more specifically in a variety of ways. For Rotter (1966), LOC and its two types, internal and external, are defined as, “when a reinforcement is perceived by the subject as following some action of his own but not being entirely contingent upon his action, then, in our culture, it is typically perceived as the result of luck, chance, fate, as under the control of powerful others, or as unpredictable because of the great complexity of forces surrounding him. When the event is interpreted in this way by an individual, we have labeled this a belief in external control. If the person perceives that the event is contingent upon his own behavior or relatively permanent characteristics, we have termed this a belief in internal control” (p. 1). Nevertheless, Lefcourt (1982) specified that “people are not totally internals or externals”; rather, “the terms internal or external control depict an individual's more common tendencies to expect events to be contingent or non-contingent upon their actions” (p. 186). More recently, and from an economics approach, McGee (2015) defined LOC as “the degree to which one believes one's action influences outcomes” (p. 184).

Perceived control has been widely examined in several psychology studies and in multiple forms of operationalization and situations (Averill, 1973; Cheng et al., 2013; Johnson et al., 2015). Research by Skinner (1996) that sought to identify the constructs that are close to perceived control (e.g., control beliefs; Skinner et al., 1988), efficacy expectations (Bandura, 1977), and attributions (Weiner, 1985) found nearly 100 terms that could be classified into a typology with two dimensions: (a) objective control, subjective control, and experiences of control and (b) agents, means, and ends of control, in which LOC is understood as a construct that leads to a means-ends relationship. Thus, according to Skinner, LOC is a generalized belief that events depend on internal or external factors.

To overcome several shortcomings in Skinner's view, Levenson (1973, 1981) replaced the one-dimensional internal/external distinction with a multidimensional approach, considering internal (I) beliefs on one side and two types of external control beliefs on the other side: chance (C) and powerful others (P). The concept of perceived control is domain-specific and transitory and concerns a particularly stressful condition, for example, health issues such as cancer (e.g., Henderson et al., 2002), alcoholism (e.g., O'Leary et al., 1976), work-related issues (e.g., Spector, 1988), or, in the case of the present article, being unemployed (e.g., Wanberg, 1997; even though a specific scale was not used in this study). The present study does not address “generalized perceived control” but rather analyzes a “domain-specific control belief” (Specht et al., 2013): unemployment locus of control.

## Locus of Control and Unemployment

General LOC has been examined in relation to coping with unemployment (Wanberg, 1997), job search strategies (Kanfer et al., 2001), and unemployment duration. For the last issue, some data have shown that the kind of locus of control predicts reemployment. Holmes and Werbel (1992) reported that individuals with a more internal LOC were more likely to be reemployed within 3 months of job loss. Ginexi et al. (2000) found that internal control beliefs were stable over a period of 12 months of unemployment, but initial internal control beliefs predicted reemployment at 6 months after sociodemographic factors were controlled for. However, no effect was found for the period after 6 months. Tiggemann and Winefield (1989) found that the LOC of school-leavers predicted unemployment 1, 3, and 5 years later. Later, Waters and Moore (2002) showed that baseline LOC predicted employment status 6 months later: Unemployed people who scored higher on powerful others and on chance were more likely to remain unemployed, whereas the opposite was true for those with a more internal LOC.

Wanberg research (1997) measured both general perceived control (the extent to which an individual views life as under one's own personal control) and situational control (perceived control over the specific situation of unemployment) of recently unemployed people. Yet neither of these kinds of LOC predicted reemployment 3 months later.

Finally, Kanfer et al.'s (2001) meta-analysis found that internal LOC was weakly related to job search behavior and somewhat more to the length of unemployment. This overall finding of internal control having more positive results is in agreement with research on general work outcomes. In their meta-analysis, Ng et al. (2006) established that internal LOC predicted outcomes such as positive task and social experiences, job satisfaction, job motivation, job performance, and career success. Finally, McGee (2015) found that whereas LOC influenced the intensity of the job search, unemployed people with internal LOC beliefs were not more likely to actually get a job as a result of that job search and thus did not reap the benefits of their more intense job search.

It has also been shown that LOC can change with the duration of unemployment and that unemployed people become more external as time passes (O'Brien and Kabanoff, 1979; Patton and Noller, 1984; Feather and O'Brien, 1986; Layton, 1987; Baubion-Broye et al., 1989; Legerski et al., 2006). The evidence for this, however, is mixed and contradictory (for a review, see Goldsmith et al., 1995) given that several authors found no such effect (e.g., Tiggemann and Winefield, 1984; Winefield and Tiggemann, 1985; Frost and Clayson, 1991). It has also been argued that the difference between LOC in the employed and the unemployed may be due to the fact that employed people shift to a more internal set of causal ascriptions (Gurney, 1981; Feather and O'Brien, 1986). The moderating or mediating dimensions that explain these variations have not yet been clearly demonstrated.

## The Need for a Specific Scale of Perceived Control of Unemployment

A large number of general scales, such as the Rotter Internal-External LOC scale (Rotter, 1966) as well as specific perceived control scales, are currently in use (for a critical review, see Furnham and Steele, 1993), but none of them were designed for unemployed people. Yet, for reasons related to their psychometric properties, specific scales seem to measure specific situations more accurately, which gives them greater validity. Thus, in reference to Rotter (1975) and Furnham and Steele (1993) concluded that specific measures function better than general LOC scales because they have been constructed in a way that is adapted to the particular domain and thus ensure greater predictive validity. These findings have been confirmed empirically. For example, studies by Hau (1995) and Wang et al. (2010) found that specific LOC scales were better suited for revealing the relations between LOC and the relative criteria from a particular field of study, and they called for the creation of specific measures rather than general ones. Therefore, for psychometric as well as ecological and contextual reasons (Lefcourt, 1992), it is necessary to construct an LOC scale that is specific to unemployment.

Despite this evidence, however, unemployment research often uses general scales to assess LOC or perceived control in unemployed people. Sometimes, perceived control is assessed with just one question, as Wanberg (1997) did to assess domain-specific control involved in unemployment. Sometimes, instruments such as the Work Locus of Control Scale (Spector, 1988) have been used, but these were clearly built from a work-based rather than an unemployment perspective. The Attributions of Employment Scale created by Gurney (1981), which can be used with both employed and unemployed people, measures very general attributions about unemployment and job seeking (e.g., “It is mainly a matter of luck whether a school-leaver gets a job or not”), but the items are not necessarily relevant for unemployed people. It is rather hazardous to assume that unemployed respondents would project their beliefs about themselves into their responses if the items are not formulated to ensure that this will be the case.

Furthermore, several authors have expressed a general call for more domain-specific assessments of LOC and perceived control (e.g., Lefcourt, 1982; Paulhus, 1983; Spector, 1988; Fournier, 2002). There is clearly a need for a scale that can assess perceived control in unemployment, given that this situation remains a key issue in contemporary society. Do unemployed people believe that they have some influence over their specific situation of having to find a job, or do they think finding a job depends on chance or on some powerful administration, family, friends, or employers? What are the outcomes of these different attitudes in terms of coping strategies, mental health, well-being, and job search results (job opportunities, jobs applied for, jobs offered, duration of unemployment, quality of employment, satisfaction with employment, etc.)? All these research questions, which are related to issues of active labor market policies, should be studied further with scales that are specifically designed to measure perceived control in the case of unemployment.

As previously mentioned (Pignault and Houssemand, 2017), the LOC variable needs to be studied further to gain a more fine-grained understanding of unemployment perceptions, coping, and personal consequences (psychological and professional ones). In addition, as prescribed, a correct evaluation of LOC needs to be contextualized. Thus, and even if other psychological variables are linked with unemployment, without a precise and objective measure of unemployment locus of control, it is impossible to understand and analyze the nomothetic network comprised of all of these constructs.

## The Present Study

The present research was divided into four studies: three cross-sectional and one longitudinal. The goal of the first and second (cross-sectional) studies was to create and validate the Perceived Control in Unemployment Scale with Structural Equation Modeling. The goal of the third study was to confirm the link between the new scale and general locus of control. With the fourth (longitudinal) study, our objective was to understand the relations between the Perceived Control in Unemployment Scale, several other psychological dimensions, and employment status after 6 months by reanalyzing a set of data that had previously been used to predict job finding (Meyers and Houssemand, 2010). The four samples were totally distinct and independent.

The objectives, steps and practical modalities of these studies have been reviewed and validated by the experts of Luxembourg National Research Fund (FNR; CORE Projects). Modalities for data collection have been accepted by both the FNR experts and the public employment services' partners in France (Pôle emploi) and Luxembourg (ADEM). We guaranteed voluntary and anonymous participation as well as data confidentiality. Information about the study, the identities of the researchers and the guaranties mentioned above have been communicated to the participants in oral and written form. An oral and informed consent was obtained from all participants. Moreover, the *Luxembourg Agency for Research Integrity* (LARI) specifies that according to Code de la santé publique - Article L1123-7, it appears that France does not require research ethics committee [Les Comités de Protection des Personnes (CPP)] approval if the research is non-biomedical, non-interventional, observational, and does not collect personal health information. Otherwise, with regard to Luxembourg regulations, Code de déontologie médicale, Chapter 5, Article 77 of states “The experimentation on a healthy subject is admitted if it is about a person of major age able to give freely his consent.” Further text describes providing information for the consent process. Furthermore, it is not a study for the development of biological or medical knowledge, thus CNER approval is not required.

## Study 1

### Method

#### Item Development

Items for this new scale were inspired by the A-form of the Multidimensional Locus of Control Health Scales, which are composed of a three-factor structure: internal control beliefs (I) and two external control factors, chance (C) and powerful others (P) (MLCH; Wallston et al., 1978). We simply changed the stressful issue from health to unemployment. We chose this scale primarily because of its initial psychometric qualities and the fact that it is domain-specific. We first wrote the items in French. We proposed that the three sets of six newly written items would reflect three dimensions of beliefs concerning a person's control over the unemployment situation, the job search process, and job search outcomes. Items are rated on a 4-point Likert scale ranging from 0 (*absolutely disagree*) to 3 (*absolutely agree*). The complete scale in French is presented in the Appendix, along with an English translation that was not used in this study.

#### Participants

The first sample consisted of 1,009 unemployed people, 584 of whom were inhabitants of Luxembourg and 425 of France. All were French-speaking. Participants were 38.59 years old on average (*SD* = 11.27); 48.4% were men and 51.6% were women; 55.0% of them had been unemployed at least once previously, and less than half of them (44.6%) had been unemployed for less than 6 months this time. Concerning level of education, 7.2% of the respondents had no academic certificate, 30.5% had an end-of-schooling equivalent, 41.5% had professional qualifications, and 20.8% had a university diploma. Finally, 93.5% of the participants had already worked for a mean duration of 14.92 years (*SD* = 11.41). All participants voluntarily responded at state employment centers in France and Luxembourg.

#### Measure

A paper-pencil questionnaire consisting of the 18 items from the Perceived Control in Unemployment Scale and some demographic and socioeconomic questions was given to the unemployed people in the first sample.

### Results

In order to validate the structure of the Perceived Control in Unemployment Scale, an item analysis was conducted by applying both classical true score theory (psych package in the R software) and item response theory (Excel tools using the eirt add in; Valois et al., 2011). These tests showed that the three LOC dimensions measured by the scale had acceptable internal consistency indicators that were close to the original scale, varying between 0.67 and 0.77 (in our study: alpha-LOC-internal = 0.65, alpha-LOC-chance = 0.71, alpha-LOC-powerful = 0.65). These reliability indicators might seem somewhat weak, but psychometric studies have shown that alpha is highly sensitive to the number of items, and the numbers were small in this study for each of the LOC scales (Cortina, 1993). Moreover, the logistic curves of the majority of these 18 items corresponded to our expectations for the IRT analysis. For example, the item, “It is my own behavior that determines how quickly I find a job” was perfectly adjusted to the latent factor (LOC-I) and followed a logistic curve (χ^2^ = 6.742, *df* = 30, *p* = 1.000). However, two items, “It is my own fault that I am unemployed” (LOC-I) and “Employers control my professional life” (LOC-P), were modestly adjusted to their respective latent factors and presented low psychometric qualities (in particular, low item-scale correlations; *r*_*it*_ = 0.145 and *r*_*it*_ = 0.228, respectively). Therefore, we removed these items from the final Perceived Control in Unemployment Scale, thus increasing the new alpha coefficients (see Table 1).

**Table 1:** Loadings of the four EFA factors of the perceived control in unemployment scale and Cronbach's alphas for each dimension.

	**F1**	**F2**	**F3**	**F4**
9. Luck plays a big part in determining how soon I will find a job.	0.792			
11. Job finding is largely a matter of good fortune.	0.854			
4. Most of the things that affect my job search happen by chance.	0.478			
16. If it's meant to be, I will become unemployed again.		0.754		
2. No matter what I do, if I am going to become unemployed, I will.		0.603		
15. No matter what I do, I'm likely to become unemployed again.		0.665		
3. Being in regular contact with the administration office is the only way for me to find a job.			0.682	
18. I can only do what the administration tells me to do in order to find a job.			0.613	
14. I can find a job if others have searched for me.			0.477	
5. If I want to find a job, I should consult a professional.			0.503	
7. My family has a lot to do with my finding a job or staying unemployed.			0.237	
6. I am in control of the way I look for a job.				0.403
12. The main things that affect my ability to find a job are what I do myself.				0.607
13. If I take care, I can avoid being unemployed again.				0.518
17. If I take the appropriate actions, I can find a job.				0.736
1. It is my own behavior that determines how quickly I find a job.				0.559
Alphas	0.72	0.77	0.65	0.69

To confirm these preliminary results, we computed exploratory factor analyses (EFAs, ML, oblimin rotation) on the set of 16 retained items with the software Mplus 7.3 (Muthén and Muthén, 2010). A first model with three factors conforming to theoretical expectations had some low fit indices (χ^2^ = 944.219, *df* = 75, RMSEA = 0.107, CFI = 0.818, TLI = 0.708, SRMR = 0.064). However, a model with four latent factors showed a better and acceptable fit (χ^2^ = 288.827, *df* = 62, RMSEA = 0.060, CFI = 0.952, TLI = 0.908, SRMR = 0.034; see Table 1). The correlation matrix for the latent factors in this analysis is presented in Table 2. Factor 1 was moderately and positively correlated with Factors 2 and 3, whereas Factors 2 and 3 had positive but small correlations with each other (intensity correlation guidelines by Cohen, 1988). However, Factor 4 was not correlated with any other factor in this analysis. According to previous research, especially the structure of the Multidimensional Locus of Control Health Scales (Wallston et al., 1978) and the correlations between factors, the first and second subfactors can be interpreted as two different but linked chance dimensions of locus of control. Using Rotter's definition of the external locus of control construct (Rotter, 1966) and the wording of the items, the first factor can be understood as a luck subdimension of locus of control, whereas the second one refers to a fate subdimension. The third factor corresponds to the powerful others dimension of locus of control and, finally, the fourth factor represents the internal dimension of locus of control.

**Table 2:** Correlations between latent factors from an EFA of the Perceived Control in Unemployment Scale and Cronbach's alphas for each dimension.

	**F1**	**F2**	**F3**	**F4**	**α**
F1. Chance 1	–				0.72
F2. Chance 2	0.241^*^	–			0.77
F3. Powerful others	0.298^*^	0.131^*^	–		0.65
F4. Internal	−0.056	−0.039	−0.031	–	0.69

* *p < 0.05*.

## Study 2

### Aim of the Study

The main objective of Study 2 was to confirm the structure of the perceived control in unemployment scale by testing its construct validity. Based on Levenson's theory of locus of control (Levenson, 1973, 1981), the Multidimensional Locus of Control Health Scales structure (Wallston et al., 1978), and the results of Study 1, CFAs were used to compare our empirical data with the locus of control theory. Thus, Study 2 was part of the process of validating the scale.

### Method

#### Participants

The second sample consisted of 831 unemployed people, 626 of whom were inhabitants of Luxembourg and 205 of France. All were French-speaking. Participants were 34.87 years old on average (*SD* = 11.20); 44.9% were men and 55.1% were women; 58.9% of them had been unemployed at least once previously, and more than half of them (55.1%) had been unemployed for less than 6 months this time. All participants voluntarily responded at state employment centers in France and Luxembourg.

#### Measure

A paper-pencil questionnaire consisting of the 16 items from the final Perceived Control in Unemployment Scale (see Study 1) and some demographic and socioeconomic questions was given to the unemployed people in the second sample.

### Results

**Figure 1:**
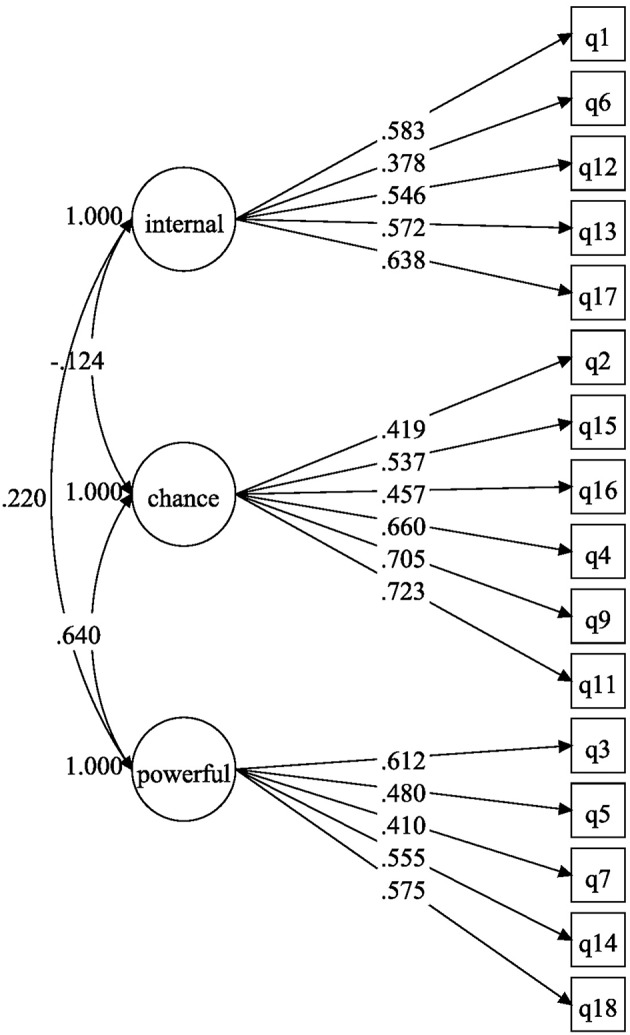
Confirmatory three-factor model of the Perceived Control in Unemployment Scale (Internal, Powerful Others, and Chance dimensions).

**Figure 2:**
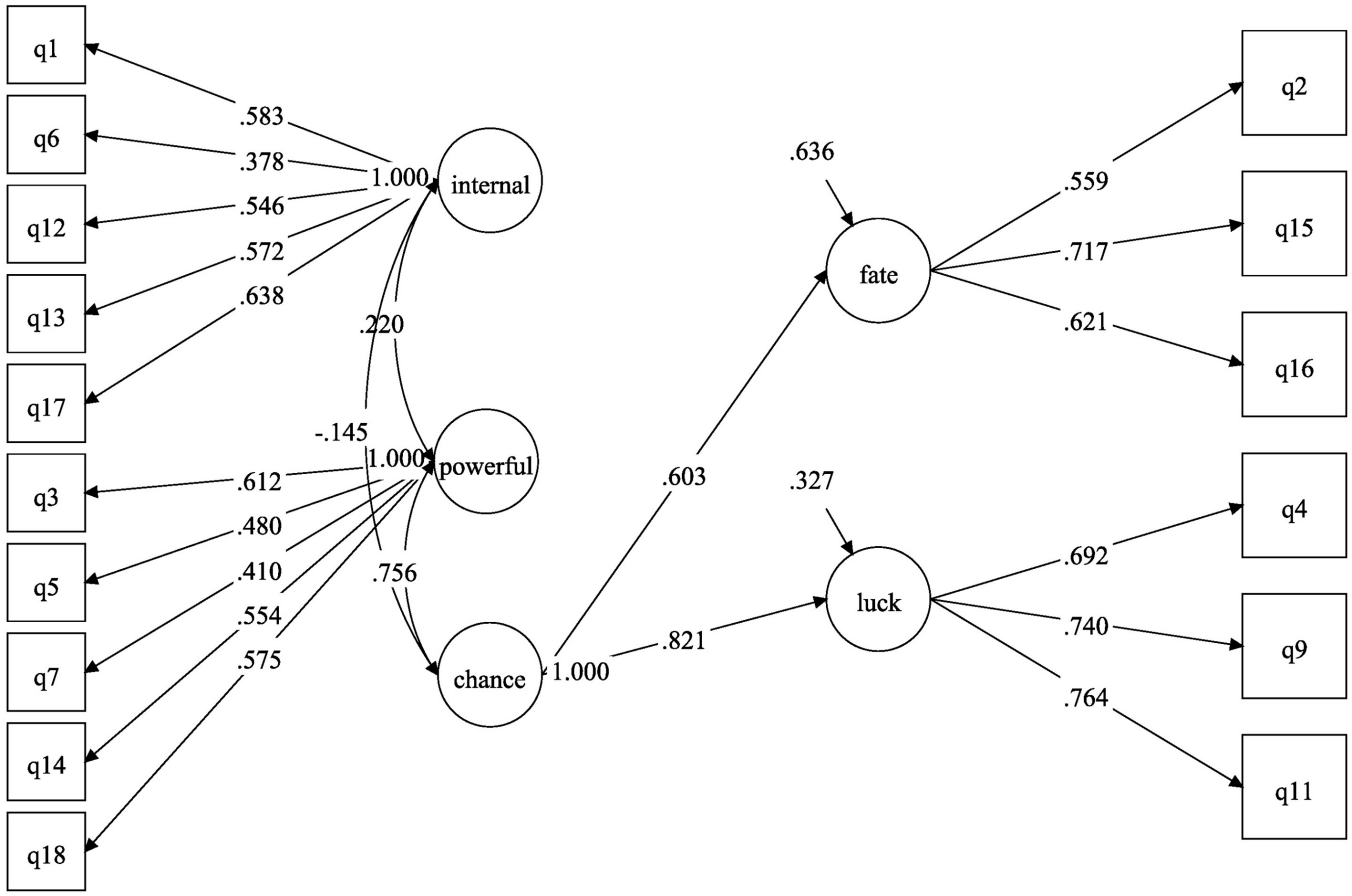
Confirmatory four-factor model of the Perceived Control in Unemployment Scale (Internal, Powerful Others, and Chance dimensions, plus the Luck and Fate subdimensions).

The previous EFA results were then confirmed by confirmatory factor analyses (CFAs) implemented in Mplus 7.3. The three-factor model of the Perceived Control in Unemployment Scale showed a rather poor fit (χ^2^ = 506.33, *df* = 101, RMSEA = 0.069, CFI = 0.881, TLI = 0.859, WRMR = 1.69), whereas the four-factor model showed that the empirical data were consistent with the four-factor structure of the scale (χ^2^ = 307.17, *df* = 99, RMSEA = 0.050, CFI = 0.939, TLI = 0.926, WRMR = 1.29; see Table 3). Given the chi-square test's known oversensitivity to sample size, minor deviations from normality, and minor model misspecifications, model fit is usually assessed with sample-size-independent fit indices such as the Comparative Fit Index (CFI), the Tucker-Lewis Index (TLI), and the Root Mean Square Error of Approximation (RMSEA). According to conventional rules of thumb (Hu and Bentler, 1999; Kline, 2011), acceptable and excellent model fit are indicated by CFI and TLI values greater than 0.90 and 0.95, respectively, and by RMSEA values smaller than 0.08 and 0.06, respectively. Thus, although the CFI and TLI for the four-factor model were somewhat low, the RMSEA was excellent. Figures 1, 2 show the models from the confirmatory tests. The first model, which we named the three-factor model, was composed of an internal dimension, a powerful others dimension, and a chance dimension. The second model, which we named the four-factor model, described the same dimensions, but the chance dimension was composed of the fate and luck subdimensions.

**Table 3:** Goodness-of-fit indices resulting from the CFA of the Perceived Control in Unemployment Scale.

	***χ^2^***	***df***	**CFI**	**TLI**	**RMSEA (90% CI)**	**WRMR**
Three-factor model	506.33	101	0.881	0.859	0.069 (0.064–0.076)	1.69
Four-factor model	307.17	99	0.939	0.926	0.050 (0.044–0.057)	1.29

*χ^2^, Chi-square; df, Degrees of Freedom; CFI, Comparative Fit Index; TLI, Tucker-Lewis Index; RMSEA, Root Mean Square Error of Approximation; CI, Confidence Interval; WRMR, Weighted Root Mean Square Residual*.

### Discussion on the Structural Perceived Control in Unemployment Scale

Although it was necessary to consider a four-factor model to obtain fit indices close to those commonly accepted in the literature, the structure of the Perceived Control in Unemployment Scale was in fact very close to the one we proposed. The LOC-C dimension required us to take into account two different first-order dimensions, but the CFA results showed that the scale did measure the three distinct factors (i.e., internal, chance, and powerful others), which were used in the third and fourth studies below. This differentiation of LOC-C into two dimensions that were strongly intercorrelated may have been due to the syntax and the formulation of the items. The syntax may have increased the apparent correlations between Items 4, 9, and 11. In fact, in the French version of the scale, the term “chance” is used in each of these three items, which translates into several ideas in English such as “chance,” “good fortune,” and “luck.” Thus, using the English version should eliminate this linguistic issue and enable us to directly model the scale with three factors. In the end, we concluded that the items from Factors 1 and 2 corresponded to the chance dimension of the scale, those from Factor 3 to the powerful others dimension, and those from Factor 4 to the internal dimension. These results were confirmed by the correlations calculated between these different factors, whose values were similar to those from previous studies (Wallston et al., 1978; Levenson, 1981). Factors 1 and 2 were strongly intercorrelated (*r* = 0.495) and were also highly correlated with Factor 3 (*r* = 0.620 and *r* = 0.456, respectively). A second-order factor derived from Factors 1 and 2 was strongly correlated with Factor 3 (*r* = 0.756). Finally, Factor 4 had low or very low correlations with all other dimensions in the model (intensity correlation guidelines by Cohen, 1988). These results permitted us to conclude that Factors 1 and 2 were two subdimensions of the LOC-C and Factors 3 and 4 represented the LOC-P and LOC-I, respectively. Thus, the final set of 16 items could be considered to comprise a valuable Perceived Control in Unemployment Scale.

## Study 3

### Aim of the Study

The main purpose of Study 3 was to confirm the link between the Perceived Control in Unemployment Scale and the general locus of control measure. Thus, we aimed to demonstrate the convergent validity of the new scale and its specificity regarding the general locus of control scale.

### Method

#### Participants

The third sample consisted of 141 unemployed people, all of whom were French-speaking inhabitants of Luxembourg. Participants were 42.75 years old on average (*SD* = 9.97); 61.0% were men and 39.0% were women; 57.9% of them had been unemployed at least once previously, and the majority of them (50.4%) had been unemployed for less than 6 months this time. All participants voluntarily responded at state employment centers in Luxembourg.

#### Measure

Participants were administered a paper-pencil questionnaire consisting of the 16 items from the final Perceived Control in Unemployment Scale (see Study 1), the 24 items from Levenson's I, P, and C scales (Levenson, 1973), and some demographic and socioeconomic questions. The validated French version of Levenson' scales were used (Rossier et al., 2002).

### Results

The structures of both locus of control questionnaires (perceived control in unemployment and general perceived control) were statistically confirmed in the present sample of participants by using the Lavaan package in the R software. Thus, it was possible to verify the convergent validity of the Perceived Control in Unemployment Scale by computing correlations between the two sets of perceived control factors. The results were very close to those from Wallston et al. study (1978) and confirmed the links between the dimensions of the two scales (see Table 4).

**Table 4:** Correlations between the latent factors of the Perceived Control in Unemployment Scale and Levenson's I, P, and C scales (Levenson, 1973).

Chance - C	0.739^***^
Powerful others - P	0.397^***^
Internal -I	0.676^***^

*** *p < 0.001*.

### Discussion

The convergent validity analysis of the Perceived Control in Unemployment Scale confirmed the links between its specific locus of control scale and the general perceived control scale. The correlations between the three locus dimensions were very close to previous results in other fields. Thus, the links that Wallston et al. (1978) identified between the Health Locus of Control Scale and Levenson's I, P, and C scales (respectively: Intern, 0.567; Powerful Others, 0.275; Chance, 0.799) were very similar to the correlations found in the present study and confirmed the validity of the new scale developed here.

## Study 4

### Aim of the Study

Based on a set of data that had previously been used to predict job finding (Meyers and Houssemand, 2010), the main goals of Study 4 were (a) to analyze the links between the Perceived Control in Unemployment Scale and several other psychological dimensions related to the unemployment situation and (b) to use a longitudinal approach to confirm that the scale could be used to predict employment status after 6 months. Based on rare research on profiling of the unemployed, psychological dimensions that predict and prevent long-term unemployment (Wanberg and Marchese, 1994; Houssemand and Meyers, 2011; Houssemand et al., 2014), and literature on the detrimental effects of unemployment (Paul and Moser, 2009) and coping with unemployment (Leana and Feldman, 1992; Leana et al., 1998; Boswell et al., 2012), a selection of variables that potentially impact unemployment duration and reemployment were selected and used as determinants of employment status after 6 months.

Because Judge et al. (2002) concluded that in fact locus of control, self-efficacy, and neuroticism are all markers of the same higher order construct, and Ajzen (2002) argued that locus of control and self-efficacy together described a broader construct equivalent to “perceived behavioral control,” one can imagine significant relationships between the dimensions of LOC and various self-perception variables (how one sees oneself among others as a candidate for a job, which has some influence on how one is seen by important actors in the job-seeking process, measured here as self-esteem, self-efficacy, and core-self-evaluations). In the same vein, because perceived control has been studied specifically in relation to coping with unemployment (Wanberg, 1997), a relationship between the two concepts would be reasonable.

Thus, this step of the research contributed to our ability to assess the discriminant validity of the scale and the predictive validity of the Perceived Control in Unemployment Scale.

### Method

#### Participants

The participants in the fourth study were recruited from the state employment agency in Luxembourg. The sample was composed of 384 newly registered unemployed people (58.8% men; 41.2% women). We recruited participants with no known previous experience with unemployment so that prior memories of joblessness could not affect the results. The majority of respondents (61.4%) had been unemployed for less than 6 months.

#### Measures

A computer-based, user-friendly assessment tool was used to administer the questionnaires. No computer skills were necessary to fill out the questionnaire because the questions were administered on tablets, which allowed the keyboard to be hidden and a stylus to be used for answering. In addition to the Perceived Control in Unemployment Scale, several psychological dimensions were measured at the beginning of unemployment to evaluate convergent and discriminant validity with known constructs and to serve as control variables. All dimensions were measured with French versions of published questionnaires. After 6 months, the 175 subjects in the sample who were still unemployed were invited to fill out the Perceived Control in Unemployment Scale again. Ninety-seven subjects (60.6%) completed the questionnaire a second time. People who had found employment during the 6 months could not be assessed because they were no longer at the employment agencies, and previous trials have shown that sending surveys by post would have resulted in negligible return rates.

*Self-esteem* was assessed with the Rosenberg Self-Esteem Scale (RSES; Rosenberg, 1965), a 10-item scale largely used for assessing general self-esteem. Items are rated on a 4-point scale ranging from 0 (*strongly disagree*) to 3 (*strongly agree*). We used the validated French version (Chambon et al., 1992).

*Self-efficacy* was measured with the General Self-Efficacy Scale (GSES; Sherer et al., 1982). Items are rated on a 5-point scale ranging from 0 (*absolutely disagree*) to 4 (*absolutely agree*). We used the French version of the questionnaire (Chambon et al., 1992).

*Core self-evaluations* (Judge et al., 2002) are an aggregation of self-esteem, neuroticism, self-efficacy, and internal locus of control. This concept has already been studied in relation to job seeking, where it was linked to persistence in job seeking and reemployment. This construct was measured with the 12-item Core Self-Evaluations Scale (CSES; Judge et al., 2003). Items are rated on a 5-point scale ranging from 1 (*strongly disagree*) to 5 (*strongly agree*). The scale was adapted from the original by applying a back-translation process.

*Social skills* were assessed with the seven-item Social Skills Scale (SSS; Ferris et al., 2001). Items are rated on a 7-point Likert scale ranging from 1 (*strongly disagree*) to 7 (*strongly agree*). The scale was adapted from the original by applying a back-translation process.

*Coping* was measured with the 15-item Cybernetic Coping Scale (CCS; Guppi et al., 2004), a shortened version of the 20-item and 40-item versions (Edwards and Baglioni, 1993). Coping is considered to consist of five different types: Change the Situation, Accommodation, Devaluation, Avoidance, and Symptom Reduction. Items are rated on a 5-point scale ranging from 0 (*do not use at all*) to 3 (*use very much*). The scale was adapted from the original by applying a back-translation process.

*Social support* was measured with the six-item Social Support Questionnaire (SSQ6; Sarason et al., 1983, 1987; Rascle et al., 2005 for the French version). This scale assesses social support as well as satisfaction with this support. On the social support scale, participants could indicate from 0 to 9 support persons. Items are rated on a 6-point scale ranging from 1 (*very unsatisfied*) to 6 (*very satisfied*).

*Psychological distress* was measured with the 12-item General Health Questionnaire (GHQ12; Goldberg, 1972), a widely used scale for evaluating lack of well-being. Items are rated on a 4-point scale ranging from 0 to 3, with anchors that differ across questions. We preferred this Likert scaling to the traditional bimodal scaling (0-0-1-1) advocated by Goldberg (1972) because it enabled a better consideration of even slight differences in participants' answers. We used a French translation of the questionnaire using the Salama-Younes et al. method (2009).

*Social anxiety* was measured with the 24-item Liebowitz Social Anxiety Scale (LSAS; Liebowitz, 1987; Bouvard and Cottraux, 2002 for the French version). For feasibility reasons, we only used the Fear of Performance Scale (Fresco et al., 2001). Items are rated on a 4-point scale ranging from 0 (*none*) to 3 (*severe*).

*Perceived stress* was measured with the 14-item Perceived Stress Scale (PSS; Cohen et al., 1983; Bruchon-Schweitzer, 2002). Items are rated on a 5-point scale ranging from 0 (*never*) to 3 (*very often*).

*Depression* was measured with the 20-item Center for Epidemiologic Studies Depression Scale (CES-D; Radloff, 1977), a widely used instrument. Items are rated on a 4-point scale ranging from 0 (*rarely or none of the time*) to 3 (*most or all of the time*). We used the French adaptation of the questionnaire (Führer and Rouillon, 1989).

In addition to these psychometric scales, demographic and socioprofessional data were also collected with the same survey tool or anonymously collected from the unemployment records of participants so that we had additional control variables: age, sex, marital status (alone vs. in a couple), nationality (Luxembourgish vs. Foreigner), initial training (general vs. technical cursus), profession (executive, employee, or worker), present income, and loans to be reimbursed (≤ 500 €, 501–1000 €, 1001–2000 €, or > 2000 € for each of the two financial variables). Finally, the dependent measure was employment status after 6 months. This information was also extracted anonymously from participants' unemployment records.

### Results

#### Intercorrelations Between Psychological Variables

Discriminant analyses were computed further as can be seen in the multiple bivariate correlations shown in Table 5. The data showed that LOC-I was significantly positively correlated with self-esteem, core self-evaluations, social skills, change situation coping, accommodation coping, symptom reduction coping, social support, and satisfaction with social support. It was significantly negatively correlated with psychological distress and perceived stress. LOC-C was significantly positively related to avoidance coping, psychological distress, social anxiety, perceived stress, and depression. It was negatively related to self-esteem, self-efficacy, core self-evaluations, social skills, social support, and satisfaction with social support. Finally, LOC-P was positively related to social anxiety, perceived stress, and depression and was negatively correlated with self-esteem, self-efficacy, core self-evaluations, social support, and satisfaction with social support.

**Table 5:** Intercorrelations between Psychometric Variables at the Beginning of Unemployment (Cronbach alphas on the diagonal and in italic).

		**1**	**2**	**3**	**4**	**5**	**6**	**7**	**8**	**9**	**10**	**11**	**12**	**13**	**14**	**15**	**16**	**17**	**18**
1	Control - I	*0.68*																	
2	Control - C	−0.07	*0.73*																
3	Control - P	0.07	0.36^***^	*0.59*															
4	Self-esteem	0.16^**^	−0.32^***^	−0.16^**^	*0.82*														
5	Self-efficacy	0.08	−0.32^***^	−0.16^**^	0.58^***^	*0.83*													
6	Core self-evaluations	0.20^***^	−0.28^***^	−0.15^**^	0.71^***^	0.67^***^	*0.78*												
7	Social skills	0.19^**^	−0.11^*^	−0.02	0.25^***^	0.37^***^	0.34^***^	*0.83*											
8	Change situation coping	0.18^***^	−0.04	0.01	0.14^**^	0.21^***^	0.15^**^	0.38^***^	*0.64*										
9	Accommodation coping	0.19^***^	−0.03	0.02	0.14^**^	0.26^***^	0.17^**^	0.31^***^	0.56^***^	*0.67*									
10	Symptom reduction coping	0.13^*^	−0.10	−0.12	−0.02	−0.01	−0.02	0.18^***^	0.33^***^	0.33^***^	*0.57*								
11	Devaluation coping	0.01	0.12^*^	−0.03	−0.01	−0.08	−0.05	0.09	0.16^**^	0.24^***^	0.46^***^	*0.71*							
12	Avoidance coping	−0.03	0.20^***^	0.03	−0.22^***^	−0.27^***^	−0.26^***^	−0.05	0.04	0.14^**^	0.42^***^	0.62^***^	*0.78*						
13	Social support	0.14^**^	−0.16^**^	−0.15^**^	0.19^***^	0.23^***^	0.26^***^	0.26^***^	0.14^**^	0.09	0.12^*^	0.05	−0.04	*0.91*					
14	Satisfaction social support	0.15^**^	−0.18^**^	−0.14^**^	0.28^***^	0.35^***^	0.29^***^	0.18^**^	0.07	0.12^*^	0.01	0.02	−0.11^*^	0.39^***^	*0.92*				
15	Psychological distress	−0.12^*^	0.15^**^	0.09	−0.47^***^	−0.26^***^	−0.49^***^	−0.12^*^	0.07	0.04	−0.01	−0.08	0.01	−0.15^**^	−0.18^***^	*0.86*			
16	Social anxiety	−0.03	0.30^***^	0.21^***^	−0.32^***^	−0.36^***^	−0.38^***^	−0.25^***^	−0.06	0.05	−0.05	0.04	0.17^**^	−0.22^***^	−0.09	0.20^***^	*0.94*		
17	Perceived stress	−0.15^**^	0.28^***^	0.16^**^	−0.62^**^	−0.57^***^	−0.69^***^	−0.30^***^	−0.15^**^	−0.11	0.01	0.07	0.27^***^	−0.31^***^	−0.31^***^	0.56^***^	0.36^***^	*0.75*	
18	Depression	−0.07	0.32^***^	0.26^***^	−0.66^***^	−0.55^***^	−0.68^***^	−0.22^***^	−0.06	−0.07	−0.01	0.03	0.20^***^	−0.30^***^	−0.34^***^	0.59^***^	0.42^***^	0.73^***^	0.91

* p < 0.05;

** p < 0.01;

*** *p < 0.001, (two-tailed)*.

#### Change in the Perception of Control After 6 Months of Unemployment

The scales for measuring perceived control were completed by 97 people who remained unemployed after 6 months. The results of a repeated-measures MANOVA are presented in Table 6 and showed no significant differences on the scales for measuring perceived control after a period of 6 months of unemployment. In order to check whether these stable results could have been produced by a difference between unemployed respondents and non-respondents, we tested differences in all the demographic and socioprofessional variables mentioned above for both groups. The only variable that showed a significant difference (χ^2^ = 6.43, *df* = 1, *p* < 0.05) was the measure of whether the participant was receiving unemployment benefits because more people who received payments responded. This difference could influence or account for stability in the perceptions of control after 6 months. If respondents received benefits and non-respondents did not, and if the people who received employment benefits were more likely to have internal LOCs, this could counterbalance a possible general shift toward externality in the entire unemployed sample.

**Table 6:** Measures of perceived control at the beginning and after 6 months of unemployment (mean and standard-deviation).

	**0 months**	**6 months**	***rtt***
All subjects	*N* = 384	*N* = 97	
Control I	9.7 (2.4)	9.2 (2.6)	0.59
Control C	6.7 (2.8)	7.4 (3.0)	0.61
Control P	6.3 (2.5)	6.1 (2.2)	0.48
**MANOVA repeated measures**	**0 to 6 months (*****N*** **=** **97)**		***F***_**(1, 95)**_
Control I	9.2 (2.3)	9.2 (2.6)	0.01 (*p* = 0.93) *ns*
Control C	6.9 (2.7)	7.4 (3.0)	2.70 (*p* = 0.10) *ns*
Control P	6.5 (2.5)	6.1 (2.2)	3.20 (*p* = 0.08) *ns*

*N, sample size; ns, non significant; rtt, test-retest correlation*.

To test this hypothesis, the three perceptions of control measured at the beginning of unemployment were compared between the unemployed people who responded and those who did not respond at 6 months and showed no significant differences. In addition, we tested whether LOC-I, LOC-C, and LOC-P at the first assessment were related to whether or not participants received unemployment benefits during the following 6 months. This relation was not significant for LOC-I, LOC-C, or LOC-P. Thus, this stability in perceptions of control after 6 months did not result from a bias that was due to differences between respondents and non-respondents at 6 months.

#### Validity of Perceived Control in Predicting Unemployment Duration

Data on the employment status of the sample were provided by the employment agencies 6 months after the assessment, when 197 (51.3%) participants had yet to find a job, and there were 12 for whom no more information was available in the national databases (certainly including persons who had left the country or who were working abroad). After 6 months, 70 unemployed people had joined an active labor market program, meaning that they did state-subsidized work (working for the State or “contrat aidé” with private companies) but were still considered unemployed. They also often stopped looking for regular, unsubsidized jobs while in this program, which in most cases lasts for 1 year. To calculate predictive validity, we excluded them from our sample because of the ambiguous nature of these programs with regard to employment status. Finally, 105 participants were categorized as unemployed people.

Using the statistical software R, we computed a logistic regression in which we predicted employment status after 6 months from the psychometric scales, adjusting for all socioprofessional variables (age, sex, marital status, nationality, initial training, profession, present income including unemployment allowance if received, and debts to be reimbursed) and all psychometric variables. The non-numeric variables were introduced by the GLM statistical process as a dummy variable for which one modality is coded 1 and the others are coded 0. The results of the logistic regression are shown in Table 7. The only scales that predicted employment status significantly after 6 months of unemployment were the three perceived control scales. For the latter, employed people scored higher on LOC-I and LOC-P than those who were unemployed, whereas the unemployed scored higher on LOC-C.

**Table 7:** Logistic regression analysis for predicting employment status after 6 months.

	**Unemployed (*****N*** **=** **105)**		**Employed (*****N*** **=** **197)**				
**Construct**	***M***	***SD***	***M***	***SD***	***p***	**Odds ratio**	**95% CI**
Control I	9.1	2.4	10.2	2.2	0.003^**^	0.80	[0.68, 0.93]
Control C	7.0	2.7	6.6	2.8	0.016^*^	1.18	[1.03, 1.36]
Control P	6.2	2.4	6.4	2.5	0.013^*^	0.83	[0.72, 0.97]
Self-esteem	21.3	5.1	21.3	4.6	0.173	1.08	[0.97, 1.20]
Self-efficacy	59.5	11.1	60.3	10.9	0.484	0.98	[0.94, 1.03]
Core self-evaluations	41.5	6.4	41.8	6.2	0.861	1.01	[0.92, 1.10]
Social skills	34.7	6.7	33.4	7.3	0.057	1.06	[1.00, 1.12]
Change the situation coping	10.0	2.0	9.9	2.3	0.956	1.01	[0.84, 1.21]
Accommodation coping	9.8	1.8	9.7	2.0	0.712	1.04	[0.84, 1.29]
Devaluation coping	8.3	2.4	8.2	2.4	0.939	1.01	[0.84, 1.20]
Avoidance coping	7.2	2.8	7.5	2.6	0.102	0.86	[0.72, 1.03]
Symptom reduction coping	9.2	2.3	9.1	2.2	0.664	1.04	[0.87, 1.24]
Social support	23.1	12.3	22.9	12.9	0.314	1.02	[0.99, 1.05]
Satisfaction with social support	27.6	6.2	28.3	6.2	0.564	0.98	[0.93, 1.04]
Psychological distress	14.5	6.5	13.2	6.3	0.750	0.99	[0.93, 1.06]
Social anxiety	18.1	14.3	20.9	13.2	0.226	0.98	[0.95, 1.01]
Perceived stress	24.6	7.8	23.4	6.2	0.055	1.08	[1.00, 1.16]
Depression	16.1	9.6	15.1	9.4	0.310	1.03	[0.97, 1.10]

Adjusting for all socioprofessional and psychometric variables. Socioprofessional variables included in the model were age, sex, marital status, nationality, initial training, profession, present income including unemployment benefits if received, and loans to be reimbursed.

* p < 0.05;

** *p < 0.01, two-tailed*.

We computed a logistic regression to conduct a predictive classification of participants into employed and unemployed (Table 7). When we used the whole model, 75.8% of people could be correctly classified compared with the observed data at 6 months. With only the three control scales, 67.2% of people were correctly classified. But even the latter result is an additional 17.2% better than the 50% result that would have been found by chance.

### Discussion

Studying the links between the dimensions of the Perceived Control in Unemployment Scale and other psychological constructs that are related to being unemployed or to the length of unemployment shows the validity of this scale. In fact, even though significant links between the various LOCs and individual variables were found, these links confirm the construct validity of the scale. The correlation analyses confirm and provide more detail about certain previous results on the link between LOC and the perception of being unemployed. These analyses also confirm that there are differences in the assessment of control in unemployed people and the impact that control has on how the person experiences unemployment and on the use of strategies that a person applies to deal with and overcome it. Finally, these analyses describe a predictive model of the risk of becoming unemployed in the long term, as people with higher LOC-I and LOC-P have a greater probability of finding work after 6 months compared with people with a high LOC-C. Finally, and in contrast to certain prior studies (for a review, see Goldsmith et al., 1995), the present studies did not find any change in LOC linked to the length of time unemployed, as none of its three dimensions were affected by the length of unemployment.

## General Discussion

Responding to the call for domain-specific scales for measuring perceived control, we designed a scale for assessing the extent to which people feel as though they have control over their unemployment. On the basis of prior research by Levenson (1973, 1981), we constructed a three-dimensional scale for measuring whether people's perceptions of control were internal, left to chance, or dependent upon powerful others. A confirmatory factor analysis confirmed this three-factor structure. The correlation between LOC-C and LOC-P was shown to be moderate, which is consistent with these two dimensions as subfactors of externality. In line with prior research on similar scales (Wallston et al., 1978; Levenson, 1981), internality was not correlated with LOC-C or with LOC-P. This final result supports the view of internality and externality as independent factors (Lefcourt, 1982; Marks, 1998) and not as opposite factors that form the poles of a one-dimensional continuum.

Convergent and discriminant validity were established with several psychometric constructs that were also measured at the beginning of the unemployment period. We found that LOC-I was correlated with dimensions that are generally considered adaptive, such as self-efficacy, self-esteem, core self-evaluations, social skills, change situation coping, accommodation coping, social support, and satisfaction with social support. LOC-P and LOC-C, on the contrary, were correlated with dimensions that are less adaptive such as social anxiety, perceived stress, and depression. Altogether, bivariate analyses showed associations between internal control and several constructs that are generally considered positive and adaptive in everyday life, especially in a work environment, whereas chance control and control by powerful others are linked to dimensions that are considered unhelpful in the same contexts.

A comparison of results at the beginning and after 6 months of unemployment showed no significant changes in any of the three scales. A further investigation found that this result was probably not a consequence of bias that was due to differences between respondents and non-respondents at 6 months. These findings do not confirm observations of a growing externality or a diminishing internality with longer periods of unemployment. As past research has been mixed and contradictory on this subject, it may be the case that situational dimensions (e.g., the difficulty of finding a job in a specific labor market context or the relative generosity of social welfare) may play a moderating role in the perception of control. It may be the case that observed shifts to more externality or more internality occur primarily in social contexts involving great difficulties. For example, the unemployed people studied by Legerski et al. (2006) faced considerable problems in finding a job due to high structural unemployment, which could be linked to the observed changes in LOC. The unemployed in Frost and Clayson's (1991) sample were facing a situation in which they believed they would be hired back soon, and there were no changes in their LOC. As the labor market in Luxembourg is more favorable (high net job creation, low unemployment rate fluctuating between 4% and 5%, generous social welfare systems providing 80% of a person's previous salary as unemployment benefits for a period of 1 year), it may be the case that the pressures were not strong enough to result in significant changes (Houssemand and Meyers, 2011; Houssemand and Pignault, 2017; Pignault and Houssemand, 2018). Of course, further research should directly study the possible link between perceived and objective constraints surrounding unemployment and changes in perceived control.

We further investigated the predictive validity of the three control scales by using employment status after 6 months as the dependent variable. The three dimensions of perceived control at the beginning of unemployment predicted employment status, when we adjusted for all other dimensions of the model. Unemployed people with control perceptions reflecting internal control or control by powerful others found jobs more quickly, whereas jobless persons who perceived that chance controlled their fates remained unemployed for longer. The results for LOC-I and LOC-C are consistent with prior results, but the fact that unemployed people who scored higher on LOC-P found jobs a bit more quickly is somewhat troubling and contradicts previous findings by Waters and Moore (2002). It is true, however, that Waters and Moore used Levenson's (1973, 1981) general, domain-unspecific LOC-I, LOC-P and LOC-C scales. The result is also somewhat unexpected as we found that LOC-P was correlated with dimensions that are generally considered disadvantageous in professional contexts and job searches, such as social anxiety, depression, and perceived stress on the one side, and lower self-efficacy, self-esteem, and core self-evaluations on the other.

Several authors (e.g., Marks, 1998; Fournier, 2002) have commented on the possibility that LOC and perceived control might not constitute one or more personality traits but rather an individual response to successes and failures in life. Yet these two views are not conflicting because personal traits are not necessarily entirely stable over time as Roberts et al. (2006) demonstrated. They can change considerably over the life span, but this usually happens slowly, and they tend to interact with life contexts. Our follow-up after 6 months of unemployment showed no significant change in perceived control, which is more consistent with a trait conception. This may be due to the fact that people do not change very rapidly or that the environment does not produce enough pressure to ensure this. At the same time, we found that people who believe in the control of powerful others found jobs more quickly, showing that people may learn through observation and experience to adjust their beliefs to specific contextual requirements.

In the case of unemployment, persons may very well perceive that they must depend on others to find a job (e.g., unemployment agencies, employers, relatives, network contacts, etc.). Relying on these more or less powerful others may contribute to their success in a job search as opposed to people who do not trust anyone. This result is consistent with Levenson's (1981) statement that externality is not always bad, especially when it concerns people for whom the perception of control by powerful others is realistic. It is also consistent with results found with Wallston's MHLC scales. These results showed that for people who are not ill, the powerful others scale is unrelated to health status. But for respondents who are acutely or chronically ill (and thus more strongly concerned about their health issues in a manner that is similar to unemployed people who want to find a job), health behaviors (e.g., medical compliance) are best predicted by the belief that powerful others control health outcomes (Furnham and Steele, 1993).

Our results also provide some evidence for the distinction proposed by Rothbaum et al. (1982) between primary and secondary control. Primary control involves attempting to control events around oneself directly. Secondary control involves adapting oneself to events that are seen as uncontrollable. Job seeking implies some controllable parameters (looking for job advertisements, sending resumes to employers, etc.) and many uncontrollable ones (the job market, the policies of employment agencies, specific expectations of potential employers, etc.). Our results provide evidence that secondary control (i.e., “adapting to powerful others”) can be as successful as primary control at least in a job search.

Future studies should assess objective parameters of constraints and primary and secondary control more directly. Constraints could be assessed, for example, with respect to the unemployment rate in the region, number of jobs offered compared with the number of people looking for a job, average length of unemployment in the person's profession, statistical risk of becoming unemployed for the long term, and so forth. In any case, our results provide additional arguments to those (e.g., Lefcourt, 1982; Paulhus, 1983; Coombs and Schroeder, 1988; Spector, 1988) who consider that perceived control should be studied domain-specifically.

There are also practical implications of our scale and its related findings. First, researchers and practitioners now have at their disposal a scale that is more suited to the situation of the unemployed and can help them understand their relationship to unemployment as well as the job-search process, job-search efficacy, and (re)employment quality (Wanberg et al., 2005; Van Hooft et al., 2013). Second, the Perceived Control in Unemployment Scale may be used as a profiling tool for people who are looking for jobs (Houssemand et al., 2014) that encompasses psychological dimensions in addition to the more traditional means of profiling (Hasluck, 2004). The latter is still done only in terms of demographic, economic, and socioprofessional variables taken from the records of the unemployed: age, gender, qualifications, duration of work experience, training, nationality, family characteristics, last job, benefit amount, and so forth. In helping to profile unemployed people from the very beginning of their unemployment, measuring perceived control would contribute to the early detection of people who are at risk for long-term unemployment and to implement active labor market programs that are differentially adapted to jobless people.

## Author Contributions

All authors listed have made a substantial, direct and intellectual contribution to the work, and approved it for publication.

### Conflict of Interest Statement

The authors declare that the research was conducted in the absence of any commercial or financial relationships that could be construed as a potential conflict of interest.
