# Supplementary material for: Adaptation and Validation of the Perceived Control in Unemployment Scale
Source: Front Psychol. 2019 Feb 28;10:383. doi: 10.3389/fpsyg.2019.00383 (PMC6403124; doi:10.3389/fpsyg.2019.00383)
Supplement: Supplementary file 1 [file Image_1.pdf]

Appendix

Items of the Perceived Control in Unemployment Scale (French and English Versions)

---

1. C'est mon propre comportement qui détermine avec quelle rapidité je sors du chômage. (I)

2. Quoique je fasse, si je dois retomber au chômage je serai au chômage. (C)

3. Avoir des contacts réguliers avec l'administration est la seule manière pour moi de retrouver du travail. (P)

4. La plupart des choses qui affectent ma recherche d'emploi m'arrivent par chance. (C)

5. Si je veux trouver du travail, je devrais consulter un spécialiste de la question. (P)

6. Je maîtrise ma façon de trouver un emploi. (I)

7. Ma famille a beaucoup à voir avec le fait que je trouve un emploi ou que je reste au chômage. (P)

*8. C'est de ma propre faute si je suis au chômage. (I)**

9. La chance joue un grand rôle dans la rapidité avec laquelle je trouve du travail. (C)

*10. Les employeurs ont la maîtrise de ma vie professionnelle. (P)**

11. Le fait de trouver du travail est principalement dû à la chance. (C)

12. Pour trouver du travail, le plus important est ce que je fais moi-même. (I)

13. Si je fais attention, je peux éviter de retomber dans le chômage. (I)

14. Je trouve un travail, si d'autres personnes ont cherché pour moi. (P)

15. Quoique je fasse, je retomberai probablement au chômage. (C)

16. Je retomberai au chômage s'il doit en être ainsi. (C)

17. Si j'agis de façon appropriée, je peux trouver du travail. (I)

18. Je ne peux faire que ce que l'administration me recommande de faire pour trouver un emploi. (P)

1. It is my own behavior that determines how quickly I find a job. (I)

2. No matter what I do, if I am going to become unemployed, I will. (C)

3. Being in regular contact with the administration office is the only way for me to find a job. (P)

4. Most of the things that affect my job search happen by chance. (C)

5. If I want to find a job, I should consult a professional. (P)

6. I am in control of the way I look for a job. (I)

7. My family has a lot to do with my finding a job or staying unemployed. (P)

*8. It is my own fault that I am unemployed. (I)**

9. Luck plays a big part in determining how soon I find a job. (C)

*10. Employers control my professional life. (P)**

11. Job finding is largely a matter of good fortune. (C)

12. The main things that affect my ability to find a job are what I do myself. (I)

13. If I take care, I can avoid being unemployed again. (I)

14. I can find a job if others have searched for me. (P)

15. No matter what I do, I'm likely to become unemployed again. (C)

16. If it's meant to be, I will become unemployed again. (C)

17. If I take the appropriate actions, I can find a job. (I)

18. I can only do what the administration tells me to do in order to find a job. (P)

*: Item removed from the final Perceived Control in Unemployment Scale
